# Ecoepidemiology of *Cryptococcus* *gattii* in Developing Countries

**DOI:** 10.3390/jof3040062

**Published:** 2017-11-03

**Authors:** Patricia F. Herkert, Ferry Hagen, Rosangela L. Pinheiro, Marisol D. Muro, Jacques F. Meis, Flávio Queiroz-Telles

**Affiliations:** 1 Postgraduate Program in Microbiology, Parasitology and Pathology, Biological Sciences, Department of Basic Pathology, Federal University of Parana, Coronel Francisco Heráclito dos Santos Avenue, Jardim das Américas, 81531-980 Curitiba, Brazil; queiroz.telles@uol.com.br; 2 CAPES Foundation, Ministry of Education of Brazil, 70040-020 Brasília, Brazil; 3 Department of Medical Microbiology and Infectious Diseases, Canisius-Wilhelmina Hospital (CWZ), 6532 SZ Nijmegen, The Netherlands; f.hagen@gmail.com (F.H.); jacques.meis@gmail.com (J.F.M.); 4 Centre of Expertise in Mycology Radboudumc/CWZ, 6532 SZ Nijmegen, The Netherlands; 5 Laboratory of Mycology, Hospital de Clínicas, Federal University of Parana, 80060-000 Curitiba, Brazil; rosangela.lameirapinheiro@gmail.com (R.L.P.); laboratoriomarisol@gmail.com (M.D.M.); 6 Communitarian Health Department, Hospital de Clínicas, Federal University of Parana, 80060-000 Curitiba, Brazil

**Keywords:** *Cryptococcus*, cryptococcosis, reservoirs, developing countries, ecoepidemiology

## Abstract

Cryptococcosis is a systemic infection caused by species of the encapsulated yeast *Cryptococcus*. The disease may occur in immunocompromised and immunocompetent hosts and is acquired by the inhalation of infectious propagules present in the environment. *Cryptococcus* is distributed in a plethora of ecological niches, such as soil, pigeon droppings, and tree hollows, and each year new reservoirs are discovered, which helps researchers to better understand the epidemiology of the disease. In this review, we describe the ecoepidemiology of the *C. gattii* species complex focusing on clinical cases and ecological reservoirs in developing countries from different continents. We also discuss some important aspects related to the antifungal susceptibility of different species within the *C. gattii* species complex and bring new insights on the revised *Cryptococcus* taxonomy.

## 1. Introduction

Cryptococcosis is a systemic fungal disease caused by yeasts belonging to the *Cryptococcus neoformans*/*C. gattii* species complexes [1], affecting both immunocompetent and immunocompromised hosts and causing devastating diseases [2]. Cryptococcal meningitis is the most common mycosis associated with acquired immune deficiency syndrome (AIDS) patients with significant morbidity and mortality especially in sub-Saharan Africa, Asia, and Latin America [3]. It is estimated that approximately 225,000 new cryptococcal meningitis cases occur globally each year, the majority of which (73%) occur in sub-Saharan Africa [3].

*C. gattii* sensu lato (s.l.) is an emerging pathogen, initially considered an endemic disease, affecting patients living in tropical and subtropical zones [4]. However, over time, the geographic distribution of *C. gattii* s.l. infections expanded to temperate climate regions including Canada and the USA [4,5,6]. In addition, many ecological niches have been investigated globally in an attempt to elucidate the environmental reservoirs [7,8,9].

In this review, we describe the ecological distribution of the *C. gattii* species complex and highlight the environmental reservoirs of this pathogen in developing countries. We also discuss some important points about the antifungal susceptibility of this species complex and changes in the *Cryptococcus* taxonomy that has recently been debated among researchers and clinicians.

## 2. *Cryptococcus gattii* Species Complex Distribution in Developing Countries

**Figure 1:**
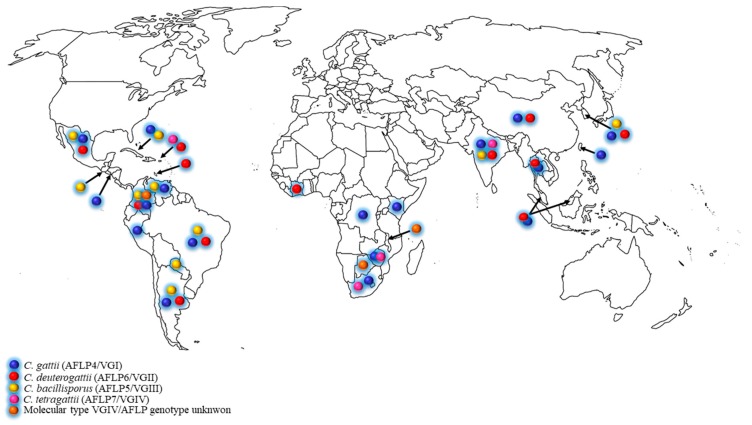
Distribution of clinical and environmental *Cryptococcus gattii* species complex in developing countries.

The *C. gattii* species complex was initially found in tropical and subtropical areas [10], but during the past two decades, the expansion to temperate climate regions was increasingly reported [5,6,11,12,13,14,15]. The ecological niches of the *C. gattii* species complex has been thoroughly investigated, and many global studies revealed that a plethora of tree species may be colonized by these pathogenic fungi [1,7,8,9,13,14,16,17,18,19,20]. The distribution of *C. gattii* species complex in developing countries is shown in the Figure 1 and Table 1. Based on these data, it became clear that the *C. gattii* species complex is not associated to a specific tree genus but that it has a predilection for plant/wood debris in general.

### 2.1. Latin America

*C. gattii* sensu stricto (s.s.) (genotype AFLP4/VGI) is a major aetiologic agent of cryptococcosis among immunocompetent patients from Brazil [25,26,27], Colombia [29], Mexico [33,34], Honduras [32], and Peru [35] and has caused pneumonia in a renal transplant patient from Argentina [21]. This pathogen has also been involved with a fatal infection in Cuba in an imported cheetah from South Africa [31]. In nature, this species was found in Psittaciformes excreta in Brazil [28] and some tree species, such as *Tipuana tipu*, *Grevillea robusta*, and *Eucalyptus* spp. in Argentina [22,23,24] and *Ficus* spp. in Colombia [30].

*C. deuterogattii* (genotype AFLP6/VGII) has been isolated from clinical samples and has been involved in meningitis, cutaneous diseases, and lung infection in immunocompetent and HIV-positive patients from Brazil [25,26,36,37,38], Colombia [29], Mexico [33,34], and French Guiana [45]. From Brazil, it was also reported causing disease in dogs [39,40]. *C. deuterogattii* is found in a variety of ecological niches, being isolated from tree detritus in Puerto Rico [46], from *Moquilea tomentosa, Plathymenia reticulata,* and *Senna sianea* in Brazil [18,41,42] and from *Eucalyptus* spp. in Colombia [30]. In addition, *C. deuterogattii* was also isolated from indoor dust from typical wooden houses in Amazonas, Brazil, and from *Guettarda acreana* trees [43,44].

The species *C. bacillisporus* (genotype AFLP5/VGIII) has been isolated from clinical samples from Colombian immunocompetent patients [29], as well as from patients in Mexico [33,34], Cuba [88], Brazil [25], Guatemala, Paraguay, and Venezuela [48]. In the environment, *Corymbia ficifolia* and *Ficus* spp. trees have been reported as reservoirs in Colombia [30,47] and *Tipuana tipu* in Argentina [23].

*C. tetragattii* (genotype AFLP7/VGIV) has been found in Puerto Rico from tree detritus [46] and the molecular type VGIV has also been found in México and Colombia, but AFLP or MLST genotyping has not been performed to differentiate *C. tetragattii* (genotype AFLP7/VGIV) and *C. decagattii* (genotype AFLP10/VGIV) from each other [33,34,35]. Some of these isolates have been recently investigated and shown to belong to *C. decagattii* rather than to *C. tetragattii* [1].

### 2.2. Africa

On the African continent, most of the literature data consists in descriptions of the *C. neoformans* species complex’s ecological distribution and clinical involvement. But only few studies have found new *C. gattii* species complex members. In South Africa, HIV-positive and HIV-negative children and adults were reported to have cryptococcosis caused by *C. gattii* s.l., but no genotyping was performed to determine the species [51,52,53]. The same holds true for Botswana [49] and Rwanda, where cryptococcal meningitis cases with *C. gattii* s.l. were found [50]. Environmental niches of *C. gattii* s.l. were investigated in Zambia, where positive samples were found in *Colophospermum mopane*, *Julbernadia globiflora*, *Eucalyptus* spp., *Brachystegia* spp., fig tree, and feces from *Hyrax midden*, but no genotyping was performed [54].

*C. gattii* s.s. (genotype AFLP4/VGI) has been recovered from HIV-positive patients, bird droppings, *Acacia xanthophloea*, and *Eucalyptus saligna* from Kenya [55], from HIV-positive patients with meningitis from Zimbabwe [57,58], D.R. Congo [32], and South Africa [56]. The species *C. deuterogattii* (genotype AFLP6/VGII) was reported causing cryptococcosis in HIV-positive patients from Ivory Coast [59]. In addition, *C. tetragattii* (genotype AFLP7/VGIV) was found to be a major cause of meningitis in HIV-positive patients in Zimbabwe [57,58] and was reported to cause cryptococcosis in patients from Botswana, Malawi, and South Africa [57,60]. A South African veterinary *C. tetragattii* (genotype AFLP7/VGIV) isolate was closely related to environmental *C. tetragattii* isolates from Colombia, Puerto Rico, and Spain [57].

### 2.3. Asia

There have been many studies performed in developing Asian countries reporting *C. gattii* s.l. causing human diseases and the ecological niche of this species complex. Unfortunately, most of the isolates were not genotyped. Chen and colleagues (2000) performed a study in Taiwan with clinical cases of cryptococcosis during the 1980s and 1990s [70]. Infections by *C. gattii* s.l. occurred in 35.6% of patients during the study period. The cryptococcosis cases included both immunocompetent and immunocompromised patients with a predominance of central nervous system (CNS) diseases [70]. In China, *C. gattii* s.l. was isolated from a surgical wound [61]. In addition, in India, it was isolated from HIV-positive and HIV-negative patients [62,63]. Environmental niches of *C. gattii* s.l. in India were recognized being tree hollows of *Syzygium cumini*, *Ficus religiosa*, *Polyalthia longifolia*, *Azadirachta indica*, *Cassia fistula*, *Mimusops elengi*, and *Cassia marginata* [64,65,66,67,68], and flowers, bark, and detritus of *Eucalyptus camaldulensis* and *E. tereticornes* [69].

*C. gattii* s.s (genotype AFLP4/VGI) was isolated from patients with meningitis in Malaysia [80,81] and India [76]; this species was also reported in clinical samples from Korean, Taiwanese, and Thai patients [79,82,83]. China has reported cryptococcosis cases in HIV-positive, HIV-negative, and immunocompetent patients [71,72,73,74,75]. The environmental source of Thai *C. gattii* s.s. is decaying wood inside a *Castanopsis argyrophylla* hollow [8], and in India this species was isolated from tree hollows [77,78].

*C. deuterogattii* (genotype AFLP6/VGII) has been isolated from chronic meningitis in Malaysia [80,81], HIV-negative patients from Korea and India [79,84,85], and immunocompetent patients in China [71,74,75]. In Thailand, this species was reported causing disease in HIV-positive and HIV-negative patients, as well as causing primary cutaneous cryptococcosis [83,86]. However, the environmental niche of this species has not been reported.

*C. bacillisporus* (genotype AFLP5/VGIII) was found causing diseases in patients from Korea [79,85], which is interesting because in Asia *C. gattii* s.s. and *C. deuterogattii* have a predominance among clinical samples. In India, the first environmental *C. bacillisporus* isolate was recovered from decaying wood of *Manilkara hexandra* [78].

*C. tetragattii* (genotype AFLP7/VGIV) was isolated in India from several clinical sources, including an HIV-positive patient with meningitis, cutaneous lesions, and granulomas in HIV-negative patients. All these isolates were genetically similar to *C. tetragattii* found in Botswana, Africa. However, only one patient had previously travelled to Egypt [87].

A hypothesis to explain the differences in geographic distribution of *C. gattii/C. neoformans* species complexes was put forward by Casadevall and colleagues (2017). These authors hypothesized that it may be attributed to the breakup of the supercontinent Pangea. The physical separation of *Cryptococcus* species complexes was an important point for its speciation [89]. In addition, it was suggested that environmental events, such as wind, ocean currents, and animals, would be involved, driving the more recent speciation of *Cryptococcus* species complexes [89]. Despite all epidemiological studies carried out, there are many countries where the presence of cryptococcal molecular genotypes has not yet been explored [90].

## 3. Antifungal Susceptibility among the *C. gattii* Species Complex

Among cryptococcal species, different antifungal susceptibility patterns have been observed. In general, the *C. gattii* species complex shows higher minimum inhibitory concentrations (MICs) of azoles than isolates from the *C. neoformans* species complex [68,91,92]. In addition, *C. gattii* s.l. clinical isolates from Taiwan showed higher amphotericin B and flucytosine MIC values than *C. neoformans* s.l. clinical isolates [70]. In Brazil, *C. deuterogattii* (genotype AFLP6/VGII) clinical isolates showed higher MIC values for flucytosine [93,94] and fluconazole than *C. neoformans* s.s. (genotype AFLP1/VNI) [93].

However, different antifungal susceptibility profiles are also present within species of the *C. gattii* species complex. *C. deuterogattii* (genotype AFLP6/VGII) has higher geometric mean MICs for flucytosine, fluconazole, voriconazole, itraconazole, posaconazole, and isavuconazole than *C. gattii* s.s. (genotype AFLP4/VGI) [95]. Lockhart and colleagues (2012) investigated the correlation of *C. gattii* species complex and its antifungal susceptibility [96]. *C. deuterogattii* (genotype AFLP6/VGII) had the highest geometric mean MIC for fluconazole, followed by *C. bacillisporus* (genotype AFLP5/VGIII), genotype VGIV (AFLP non-genotyped), while *C. gattii* s.s. (genotype AFLP4/VGI) had the lowest among species [96]. Trilles and colleagues (2012) also observed that *C. deuterogattii* isolates had higher MICs of azoles than *C. gattii* s.s. [93]. An Indian study showed that clinical and environmental *C. gattii* s.l. isolates had high fluconazole MICs [68]. However, despite these differences in antifungal susceptibility among cryptococcal species, the initial cryptococcosis therapy is the same; the clinical management changes are according to presentations and immune status, but does not consider the species involved in the disease [97].

Another important point is the phenomenon of heteroresistance, the ability of adaptation to a high concentrations of drugs, observed in *C. gattii* s.l. to itraconazole and fluconazole [98,99]. The development of heteroresistance is related to phenotypic changes, such as a decrease in cell and capsule size, low ergosterol content in the cell wall, less susceptibility to oxidative stress, and a great ability to proliferate inside macrophages [98,99]. This intrinsic mechanism present in members of the *C. neoformans/C. gattii* species complexes may contribute to a relapse of cryptococcosis during maintenance therapy [98,100]. However, the clinical importance of heteroresistance is not yet clear and requires further investigation [100,101].

## 4. The *C. gattii* Species Complex: Four Molecular Types, Five Genotypes or Five Species?

The taxonomy of the tremellomycetous yeasts has recently been revised [102,103]. Since the genus *Cryptococcus* was described, it has grown out as a highly polyphyletic one that contained more than 100 species within the orders Filobasidiales, Tremellales, and Trichosporonales [102,103]. The taxonomic revision of the genus *Cryptococcus* has been extensively discussed over the past two decades. At the 6th International Conference on *Cryptococcus* and Cryptococcosis (ICCC) debate, “*How many species and varietal states are there?*” [104], different hypotheses were discussed about the status of the *C. neoformans/C. gattii* species complex: Should the situation be kept in a “two-species division”? [105,106]. Should it be divided into six species? [107,108]. Eight? [109]. The hypotheses were supported based on different opinions about the definition of species. The first one was supported by the idea that phenetic, biological, and cladistic species concepts need to be used together to proper classify the agents of cryptococcosis, because genetic variation as shown by the molecular types does not always reflect their biological characteristics [105,106,110]. However, the second hypothesis was based on phylogenetic support that included analysis of mitochondrial, ribosomal, and nuclear genes to investigate the relationship among the various *C. neoformans* and *C. gattii* genotypes. The different genotypes clustered in six monophyletic lineages for all loci studied, suggesting that *C. neoformans* serotype A and D represent two different species and that *C. gattii* genotypes represent four individual taxa [107,108]. The third hypothesis goes a little further, considering that each genotype within *C. neoformans* and *C. gattii* has sufficient genotypic variation to be considered a different species [109]. 

Phenotypic diversity within the *C. gattii* species complex is also supporting the division of five species. Capsule and cell size showed to be variable within the complex, *C. gattii* s.s. (genotype AFLP4/VGI) had the largest capsules but smaller cells compared to the other species, while *C. deuterogattii* (genotype AFLP6/VGII) has the largest cells but smaller capsules [111]. All species in the *C. gattii* species complex have the ability to grow at 25, 30, and 35 °C, but with variable tolerance to 37 °C [1,111]. *C. deuterogattii* (genotype AFLP6/VGII) has the highest thermotolerance to 37 °C, while *C. gattii* s.s. (genotype AFLP4/VGI), *C. bacillisporus* (genotype AFLP/VGIII), and *C. tetragattii* (genotype AFLP7/VGIV) have less growth at 37 °C than 30 °C [1,111,112]. There is no significant difference in tolerance to oxidative or osmotic stresses among species [111,112].

The understanding of genetic diversity is an important step for the discovery of previously unrecognized phenetic differences [111]. The exact moment that individuals in an ancestral species are split into progeny species is not recognized for any method of species delimitation, because this process needs time until the changes in morphology, mating behavior, or gene sequences may be recognized in the progeny species [113]. Phylogenomic analyses calculated the time since divergence of the *C. neoformans* species complex and the *C. gattii* species complex to be ~34 million years ago (mya) [114]; the divergence between *C. deuterogattii* (AFLP6/VGII) and the other species of the *C. gattii* species complex occurred ~12 mya [114,115]; and the divergence of *C. neoformans* (AFLP1/VNI/AFLP1; VNII/AFLP1A/1B) and *C. deneoformans* (VNIV/AFLP2), ~24 mya [115]. The divergence among the species within the *C. neoformans/C. gattii* species complexes occurred recently, and will most likely continue as an ongoing process. The occurrence of interspecies hybrids may also be attributed to the recent divergence event, because species currently hybridizing are most likely the youngest [116].

Although a revision of the cryptococcal taxonomy has been published, part of the cryptococcal research community is not fully in favor of using the ‘seven species recognition’. Some investigators believe that it will lead to taxonomic instability due to the fact that there are most likely more species present. Many points have been discussed, including the number of isolates used, the use of phylogenetic approaches for species delineation, the accommodation of hybrids in the new taxonomy, and the fact that the new names may cause confusion between the published literature and clinical practice [110]. With these points of view, Kwon-Chung and colleagues (2017) suggested the use of the “*C. neoformans* species complex” and the “*C. gattii* species complex” as an intermediate step, instead of using the seven species nomenclature, until biological and clinical relevant differences become clear [110]. Although, according to Hagen and colleagues (2017), it is important to consider the presence of different species inside the complexes to avoid delay in the clinical progress [117].

## 5. Final Remarks

Clinical and environmental occurrence of the *C. gattii* species complex is related to geographic location, which may be attributed to the (micro)climate, or even a lack of diagnosis/environmental isolation. Cryptococcosis in most developing countries is underreported and the precise burden of cryptococcosis caused by the *C. gattii* species complex is uncertain. In addition, not all clinical laboratories differentiate the pathogenic *Cryptococcus* species. In the environment, many tree species have been described as a reservoir, proving that the *C. gattii* species complex has no tree species-specific relation, and is widely spread in the environment.

*C. gattii* species complex members differ in phenotypic traits, as capsule and cell size, thermotolerance and antifungal susceptibility. Many studies have demonstrated higher MICs of azoles for members of the *C. gattii* species complex compared to the *C. neoformans* species complex. Difference in antifungal susceptibility has also been observed within the *C. gattii* species complex, with *C. deuterogattii* (genotype AFLP6/VGII) being less susceptible to azoles than *C. gattii* s.s (genotype AFLP4/VGI). However, in vitro antifungal susceptibility does not correlate to in vivo susceptibility. Clinical manifestations in patients with *C. gattii* s.l. infections tend to be more severe than *C. neoformans.* In the former, cerebral involvement causes more hydrocephalus, focal CNS signs, as well as papilledema, ataxia, hearing loss, altered mentation, and neurological sequelae. Usually, meningo-encephalitis caused by *C. gattii* s.l. is followed by higher intracranial pressures, sometimes irresponsible to multiple LPs and/or CNF shunts. Simultaneous pulmonary involvement in >50% of patients is also observed, and mass lesions (cryptococcomas) are associated to a prolonged clinical course and respond slowly to therapy.

A new taxonomy of the polyphyletic genus *Cryptococcus* has been published, including the medical important species complexes *C. neoformans* and *C. gattii*. In addition, there are different opinions about the new classification. The presence of genetic differences within the *C. gattii* species complex needs to be considered in future studies to correlate genotypic and phenotypic traits of each species to diseases clinical presentation.

## Figures and Tables

**Table 1:** *Cryptococcus gattii* species complex in developing countries.

Continent	Species	Source	Country
**Latin America**	*C. gattii* s.s.	Clinical, Environmental, Veterinary	Argentina [21,22,23,24], Brazil [25,26,27,28], Colombia [29,30], Cuba [31], Honduras [32], Mexico [33,34], Peru [35]
	*C. deuterogattii*	Clinical, Environmental, Veterinary	Brazil [18,25,26,36,37,38,39,40,41,42,43,44], Colombia [29,30], French Guiana [45], Mexico [33,34], Puerto Rico [46]
	*C. bacillisporus*	Clinical, Environmental	Argentina [23], Brazil [25], Colombia [29,30,47], Cuba [48], Guatemala [48], Mexico [33,34], Paraguay [48], Venezuela [48]
	*C. tetragattii*	Environmental	Colombia [35], Mexico [33,34], Puerto Rico [46]
**Africa**	*C. gattii* s.l.	Clinical, Environmental	Botswana [49], Rwanda [50], South Africa [51,52,53], Zambia [54]
	*C. gattii* s.s.	Clinical	D.R. Congo [32], Kenya [55], South Africa [56], Zimbabwe [57,58]
	*C. deuterogattii*	Clinical	Ivory Coast [59]
	*C. tetragattii*	Clinical, Veterinary	Botswana [60], Malawi [60], South Africa [57]; Zimbabwe [57,58]
**Asia**	*C. gattii* s.l.	Clinical, Environmental	China [61,62,63], India [64,65,66,67,68,69], Taiwan [70]
	*C. gattii* s.s.	Clinical, Environmental	China [71,72,73,74,75], India [76,77,78], Korea [79], Malaysia [80,81], Taiwan [82], Thailand [8,83]
	*C. deuterogattii*	Clinical	China [71,74,75], India [84], Korea [79,85], Malaysia [80,81], Thailand [83,86]
	*C. bacillisporus*	Clinical, Environmental	India [78], Korea [79,85]
	*C. tetragattii*	Clinical, Veterinary	India [87]

s.s.: sensu stricto; s.l.: sensu lato.
